# Soybean Growth Promotion by the Euryhaline Marine Bacterium *Cobetia amphilecti* KMM 296 Is Associated with Hormonal and Transcriptional Responses

**DOI:** 10.3390/plants15203221

**Published:** 2026-10-20

**Authors:** Yulia A. Yugay, Andrey A. Sadykov, Olesya D. Kudinova, Yulia L. Yaroshenko, Alexandra V. Fialko, Maria R. Sorokina, Egor S. Alaverdov, Elena A. Vasyutkina, Tatiana V. Rusapetova, Olga I. Nedashkovskaya, Victor P. Bulgakov, Larissa A. Balabanova, Yury N. Shkryl

**Affiliations:** 1 Federal Scientific Center of the East Asia Terrestrial Biodiversity, Far East Branch of Russian Academy of Sciences, Vladivostok 690022, Russia; 2 G.B. Elyakov Pacific Institute of Bioorganic Chemistry, Far Eastern Branch, Russian Academy of Sciences, Vladivostok 690022, Russia

**Keywords:** *Cobetia amphilecti*, euryhaline marine bacterium, plant growth-promoting bacteria, soybean, seed inoculation, cell-free culture supernatant

## Abstract

Marine bacteria remain insufficiently explored as potential plant bioinoculants. This study evaluated the plant growth-promoting potential of the euryhaline marine bacterium *Cobetia amphilecti* KMM 296 by comparing the effects of viable cells and cell-free culture supernatant on *Glycine max*. Compared with control plants, viable *C. amphilecti* cells increased seed germination by approximately 11%, while root length and root weight were more than 2.3 and 1.8 times higher, respectively. At the reproductive stage, bacterial treatment nearly doubled the seed number and increased yield per plant by approximately 2.4-fold. These changes were accompanied by increases in indole-3-acetic acid, gibberellin, and photosynthetic pigment levels. The treatment also upregulated genes associated with auxin biosynthesis and signaling, gibberellin metabolism, and root elongation by 1.58–4.97-fold. The supernatant produced weaker or intermediate responses, including increases in soybean root length, seed number, and yield. Comparative reconstruction of 23 metabolic pathways across 34 *Cobetia* genomes predicted conserved metabolic potential for auxin-related and volatile-compound metabolism, phosphate acquisition, polyamine biosynthesis, and stress adaptation. Overall, viable KMM 296 cells exerted substantially stronger plant responses than the cell-free culture supernatant, supporting further evaluation of this strain as a candidate euryhaline marine bioinoculant.

## 1. Introduction

The development of sustainable crop-production technologies requires biological approaches that support plant establishment and productivity while reducing dependence on intensive chemical inputs. Plant growth-promoting bacteria (PGPB) are promising components of such technologies because they can influence root development, nutrient acquisition, and hormonal regulation. Seed inoculation is particularly attractive as a simple means of delivering beneficial microorganisms directly to germinating seeds and emerging roots. Positive effects of bacterial inoculants have been reported in soybean and other crops [1,2,3].

A major challenge in developing bacterial inoculants is the limited predictability of their effects, which often depend on the bacterial strain, plant species, and cultivation conditions. Plant growth promotion may be mediated either by viable bacterial cells or by compounds released into the culture medium. Cell-free bacterial preparations can promote seed germination and seedling growth under both optimal and stressful conditions [4,5]. Nevertheless, direct within-study comparisons between viable PGPB inoculants and cell-free preparations derived from the same bacterial strain remain limited [6,7]. Such comparisons can assess whether a cell-free preparation retains plant growth-promoting activity and characterize how its effects differ from those of the corresponding viable-cell inoculum.

Bacteria adapted to terrestrial saline environments, including those associated with halophytic plants, have been increasingly investigated as potential bioinoculants. Studies have demonstrated that these bacteria can promote plant growth and improve salinity tolerance in heterologous terrestrial hosts [8,9]. Marine ecosystems represent another potential source of plant-beneficial microorganisms. Previous studies showed that *Salinispora arenicola* strains isolated from coral reef organisms alleviated salinity stress in wild tobacco and tomato [10,11], while *Dermacoccus* strains from deep-sea sediments promoted tomato seedling growth under saline conditions [12,13]. Although these findings demonstrate that marine bacteria can benefit terrestrial plants, current knowledge is based on a small set of bacterial taxa and plant systems and predominantly reflects experiments conducted under saline conditions. Whether marine bacteria can also enhance crop growth and productivity in the absence of experimentally imposed salt stress therefore remains poorly understood. Assessing their effects under such conditions provides a baseline for future comparisons aimed at distinguishing general plant growth-promoting activity from salinity-specific effects.

The euryhaline marine strain KMM 296, previously assigned to *Cobetia marina* and subsequently reclassified as *C. amphilecti*, exhibits broad salinity and temperature tolerance [14,15]. It can also utilize crude petroleum as the sole carbon source [14] and produces several extracellular enzymes, including alkaline phosphatases and a DegP-type protease [16,17]. Heterologous expression of its alkaline phosphatase gene (*phoA*) in tobacco enhanced plant tolerance to abiotic stress [18]. The reported ability of KMM 296 to assimilate L-proline, sodium malonate, and maltose, together with the presence of metabolic pathways characteristic of bacteria inhabiting terrestrial plant rhizospheres [14,15], indicated considerable metabolic versatility and provided a rationale for evaluating the potential growth-promoting activity of KMM 296 in a terrestrial plant system.

Soybean (*Glycine max* (L.) Merr.) is a major global source of plant protein and vegetable oil, and therefore represents an important target for sustainable yield-enhancement strategies [19]. Previous studies have shown that non-rhizobial plant growth-promoting bacteria can enhance soybean root and shoot development, nutrient acquisition, photosynthetic performance, stress tolerance, and productivity, although the magnitude of these effects varies among bacterial strains and cultivation conditions [1,2,3]. Its agronomic importance and documented responsiveness to bacterial inoculation make soybean a suitable crop for evaluating the bioinoculant potential of a euryhaline marine bacterium.

In this study, we evaluated *C. amphilecti* KMM 296 as a soybean seed inoculant in the absence of experimentally imposed salt stress by directly comparing the effects of viable bacterial cells and the corresponding cell-free culture supernatant. We tested whether the two preparations elicited different responses in seed germination, root development, plant growth and productivity, phytohormone and photosynthetic pigment levels, total phenolic content, and the expression of selected growth- and hormone-related genes. Finally, targeted comparative reconstruction of selected metabolic pathways across the genus Cobetia was used to place the experimentally observed plant responses in the context of the predicted metabolic potential of KMM 296.

## 2. Materials and Methods

### 2.1. Bacterial Strain and Culture Conditions

The marine bacterium *C. amphilecti* KMM 296, obtained from the Collection of Marine Microorganisms (G.B. Elyakov Pacific Institute of Bioorganic Chemistry, Far Eastern Branch of the Russian Academy of Sciences, Vladivostok, Russia), was used in this study. The strain was routinely maintained on Marine Agar 2216 (Difco, Franklin Lakes, NJ, USA) at 28 °C and cryopreserved at −80 °C in 20% (*v*/*v*) glycerol.

For preparation of the viable-cell suspension, a single bacterial colony was inoculated into 100 mL of LB medium (Difco, USA) in a 500-mL Erlenmeyer flask and cultured at 28 °C for 12 h with orbital shaking at 120 rpm. Bacterial cells were harvested by centrifugation at 3000× *g* for 10 min, washed twice with sterile phosphate-buffered saline (PBS; pH 7.4), and resuspended in processed uninoculated LB medium diluted to 50% (*v*/*v*) with sterile distilled water. The bacterial cell density was adjusted to 1 × 10^8^ CFU mL^−1^ based on OD_600_ using a previously established calibration curve. The bacterial suspension was used immediately after preparation.

To prepare the cell-free culture supernatant, a parallel bacterial culture grown under the same conditions was centrifuged at 10,000× *g* for 30 min at room temperature. The supernatant was carefully collected and passed through a sterile 0.22-µm membrane filter (Millipore, Burlington, MA, USA). The absence of viable bacterial cells was verified by spreading 100 µL of the filtrate onto LB agar plates and incubating them at 28 °C for 48 h. The cell-free culture supernatant was diluted 1:1 with sterile distilled water to obtain a final concentration of 50% (*v*/*v*) and used immediately.

The medium control consisted of uninoculated LB medium processed under the same conditions as the bacterial culture and diluted 1:1 with sterile distilled water.

### 2.2. Plant Material and Seed Treatment

Seeds of soybean (*Glycine max* (L.) Merr.) cv. Lira, harvested in 2024, were sourced from Agrofirma AELITA (Moscow, Russia). Seeds were surface-sterilized with 1% (*v*/*v*) sodium hypochlorite for 10 min and rinsed three times with sterile distilled water. Surface-sterilized soybean seeds were assigned to three treatment groups: (1) 50% (*v*/*v*) uninoculated LB medium (control); (2) 50% (*v*/*v*) cell-free culture supernatant; and (3) viable *C. amphilecti* cells suspended in 50% (*v*/*v*) uninoculated LB medium. Seeds were soaked in the respective treatment solutions for 48 h at room temperature in darkness and then used directly, without washing, in the experiments described below.

For the germination assay, treated seeds were placed in Petri dishes lined with sterile filter-paper discs moistened with sterile distilled water. Each dish contained 30 seeds and served as an experimental unit, with three replicate dishes used per treatment (*n* = 3). Germination percentage was determined after seven days. A seed was considered germinated when the radicle reached a length of at least 2 mm.

Root development was evaluated in a separate experiment. Following treatment, 20 seeds per treatment were individually placed in separate tubes containing 0.7% (*w*/*v*) water agar, with each tube serving as an experimental unit (*n* = 20). At 20 days after sowing, the seedlings were removed from the tubes, and the roots were carefully separated and gently blotted to remove surface moisture. Root length and root fresh weight were then determined.

A separate pot experiment was conducted to assess soybean growth, physiological and biochemical parameters, gene expression, and productivity. Following seed treatment, seeds were sown in pots containing 1 kg of commercial seedling substrate Krepysh (Fasco, Moscow, Russia), composed of high-moor peat, lowland peat, sand, and dolomite flour. According to the manufacturer’s specifications, the substrate had a pH of 6.0–7.0 and nitrogen, phosphorus, and potassium contents of 400, 1000, and 60 mg kg^−1^, respectively. No additional fertilizers were applied. Each treatment initially comprised 15 biological replicates, with one plant maintained per pot. Plants were grown in a controlled-environment chamber at 25 °C under a photosynthetic photon flux density of 150 μmol m^−2^ s^−1^ and a 16-h light/8-h dark photoperiod. Pots were randomly assigned to positions within the chamber and remained in those positions throughout the experiment. Relative humidity was not recorded. Plants were irrigated with water as required, and no additional bacterial treatments were applied. At 20 days after sowing, five plants per treatment were harvested from each independent experiment for physiological, biochemical, and molecular analyses. Each plant was processed and analyzed as a separate biological sample. Samples were either analyzed immediately or frozen in liquid nitrogen and stored at −80 °C until analysis. The remaining ten plants per treatment were grown until approximately 80 days after sowing, when mature pods were harvested separately from each plant. Seeds were removed from the pods and dried to constant weight before weighing. Plant height, pod number, seed number, and seed dry weight per plant were then determined.

### 2.3. Phytohormone Determination

Endogenous levels of indole-3-acetic acid (IAA) and gibberellic acid (GA) were determined by enzyme-linked immunosorbent assay (ELISA) following previously described procedures with minor modifications [20,21]. Five individual plants per treatment were analyzed as biological replicates (*n* = 5), with root and shoot tissues assayed separately. Fresh root or shoot tissue (0.5 g) was homogenized in 2 mL of ice-cold 80% (*v*/*v*) methanol containing 1 mmol L^−1^ butylated hydroxytoluene (BHT). The homogenates were extracted for 4 h at 4 °C with continuous shaking and then centrifuged at 5000× *g* for 8 min at 4 °C. The supernatants were collected, purified using Copure^®^ C18 solid-phase extraction cartridges (Biocomma, Shenzhen, China), and adjusted to a constant volume with the assay diluent.

IAA and GA were quantified using the Plant Indole-3-acetic Acid ELISA Kit (PD-Pl6239E) and Plant Gibberellic Acid ELISA Kit (PD-Pl6275E), respectively (Puda Scientific Co., Ltd., Wuhan, China). Calibration curves were prepared using two-fold serial dilutions over concentration ranges of 0–50 ng mL^−1^ for IAA and 0–10 ng mL^−1^ for GA_3_. Samples and standards were incubated with the corresponding antibodies in 96-well microplates at 37 °C for 30 min, followed by incubation with the enzyme-conjugated secondary antibody under the same conditions. After washing, *o*-phenylenediamine containing hydrogen peroxide was added as the chromogenic substrate. The reaction was terminated according to the manufacturer’s instructions, and absorbance was measured at 490 nm using a microplate reader. Hormone concentrations were calculated from the corresponding calibration curves, normalized to sample fresh weight, and expressed as ng g^−1^ fresh weight (FW). All samples within each tissue type were processed and analyzed under identical conditions, supporting comparisons among treatments.

### 2.4. Determination of Photosynthetic Pigments

Chlorophyll a (Chla), chlorophyll b (Chlb), and total carotenoid (Car) contents were determined spectrophotometrically according to Lichtenthaler and Wellburn [22]. Five plants per treatment were analyzed as biological replicates (*n* = 5). Fresh leaf tissue (90 mg) was homogenized in 1 mL of 96% (*v*/*v*) ethanol and incubated for 10 min at 4 °C in darkness. After centrifugation at 10,000× *g* for 10 min at 4 °C, the absorbance of the extracts was measured at 665, 649, and 470 nm using a Benchmark Plus Microplate Reader (Bio-Rad, Hercules, CA, USA), with 96% ethanol as the blank. Absorbance values were normalized to an optical path length of 1 cm.

Pigment concentrations in the extracts were calculated using the following equations:

Chla = 13.95A_665_ − 6.88A_649_

Chlb = 24.96A_649_ − 7.32A_665_

Car = (1000A_470_ − 2.05Chla − 114.8Chlb)/245

The pigment contents were expressed as μg g^−1^ FW.

### 2.5. Determination of Total Phenolic Content

Total phenolic content (TPC) was determined using the Folin–Ciocalteu colorimetric method described by Singleton et al. [23], with minor modifications. Briefly, 25 µL of plant extract was mixed with 25 µL of Folin–Ciocalteu reagent previously diluted 1:3 (*v*/*v*) with distilled water. After incubation at room temperature for 5 min, 200 µL of distilled water and 25 µL of 10% (*w*/*v*) sodium carbonate were added. The reaction mixture was incubated at room temperature for 1 h in the dark. Total phenolic content was determined in five plants per treatment (*n* = 5), using a separate leaf extract from each plant.

Absorbance was measured at 725 nm using a Benchmark Plus Microplate Reader (Bio-Rad Laboratories, Hercules, CA, USA). Gallic acid standards ranging from 0 to 200 µg mL^−1^ were processed under the same conditions and used to construct the calibration curve. TPC was calculated from the calibration curve and expressed as mg gallic acid equivalents (GAE) g^−1^ FW.

### 2.6. Expression Analysis

Total RNA was isolated from approximately 100 mg of frozen root tissue, and first-strand cDNA was synthesized as previously described [24]. Three biological replicates, each derived from an individual plant, were analyzed per treatment (*n* = 3), with three technical qPCR replicates per biological replicate. Quantitative real-time PCR (qPCR) was performed using HS-qPCR 2× SYBR Blue Master Mix (Biolabmix, Novosibirsk, Russia) on a CFX96 Real-Time PCR System (Bio-Rad Laboratories, Hercules, CA, USA). Gene-specific primers used for expression profiling are listed in Table S1. The F-box protein family gene *GmCons6* served as the reference gene for normalization. It was selected based on a comparison of its expression stability with that of four other commonly used soybean reference genes: translation elongation factor 1-beta (*GmELF1B*), cyclophilin 2 (*GmCYP2*), actin (*GmACT*), and β-tubulin (*GmTUB*), using RefFinder [25] (Figure S1). Primer-specific amplification efficiencies and coefficients of determination (*R*^2^) were calculated from standard curves generated from serial dilutions of purified PCR products and are reported in Table S1. Product specificity was verified by melting-curve analysis using CFX Manager Software version 1.5 (Bio-Rad Laboratories, USA). Relative transcript levels were calculated using the 2^−ΔΔCt^ method, with the medium control used as the calibrator [26].

### 2.7. Isolation of Introduced Cobetia amphilecti KMM 296 from Soybean Roots

To assess the persistence of culturable KMM 296 cells associated with soybean roots, bacterial recovery was performed at 20 and 80 days after sowing. Five individual plants per treatment were analyzed separately at each sampling time, with each plant serving as an experimental unit (*n* = 5). After removal from the growth substrate, the roots were gently shaken to remove loosely adhering particles and washed six times with sterile PBS. A 100-mg portion of fresh roots was transferred to a sterile tube containing 1 mL of PBS and sterile glass beads and vortexed vigorously for 2 min to detach root-associated bacterial cells. Serial dilutions of the resulting suspensions were spread on selective peptone-yeast extract agar supplemented with 6% (*w*/*v*) NaCl, 10 µg mL^−1^ oxacillin, and 15 µg mL^−1^ lincomycin. The selective conditions were chosen based on the reported halotolerance and antibiotic resistance of KMM 296 [15]. The selectivity of the medium was verified using a pure KMM 296 culture and root suspensions obtained from plants that had not been inoculated with viable bacterial cells. KMM 296 retained the ability to form colonies under these conditions, whereas no bacterial growth was detected in root washes from the control plants. Plates were incubated at 28 °C for 72–96 h.

KMM 296 produced readily distinguishable, slightly yellow colonies on the selective medium, allowing their presumptive identification. Colonies exhibiting this characteristic morphology were randomly selected from independent plates and verified by colony PCR using primers targeting the *CaAP* gene of KMM 296. PCR was performed using the primer pair listed in Table S1 and the BioMaster HS-Taq PCR Kit (Biolabmix, Novosibirsk, Russia), and representative amplification products are shown in Figure S2A. The abundance of culturable root-associated KMM 296 cells was expressed as CFU per gram of fresh root weight.

### 2.8. Prediction and Comparative Analysis of Metabolic Pathways

The comparative dataset comprised all 34 *Cobetia* genome assemblies available in the NCBI Reference Sequence database at the time of retrieval. Genome sequences were retrieved using ncbi-genome-download v0.3.0 (https://github.com/kblin/ncbi-genome-download/releases/tag/0.3.0; accessed on 16 March 2023). Inclusion in RefSeq, which applies NCBI sequence and annotation quality requirements, served as the genome-quality criterion. Both complete and draft RefSeq assemblies were retained, and no additional filtering based on assembly level or predicted pathway content was applied. The genome sequence of *C. amphilecti* KMM 296 used in the analysis is available under RefSeq accession NZ_JQJA00000000.1.

Metabolic pathways were predicted using *gapseq* v1.2 (https://github.com/jotech/gapseq/releases/tag/v1.2; accessed on 23 July 2023), a software package designed for genome-based reconstruction of bacterial metabolic capabilities [16,27]. Pathway prediction was performed using the gapseq find workflow with the default bacterial reference databases and standard homology-search parameters, including a minimum bitscore of 200 and query coverage of 75%. Pathway completeness was calculated by gapseq from the proportion of detected reactions, accounting for spontaneous reactions and reactions lacking reference sequences. Under the default gapseq criteria, a pathway was classified as present (Prediction = True) when at least 80% of its reactions were detected, or when at least 66% were detected together with additional supporting evidence, such as the presence of a key enzyme. No additional presence or completeness cutoff was imposed. Completeness values were retained to show the extent of reaction-level support for each prediction.

From all pathways assessed, 23 were manually selected for visualization based on their annotated functions and potential relevance to plant–microbe interactions. These pathways represented phytohormone and volatile-compound metabolism; phosphorus, nitrogen, and iron metabolism; polyamine metabolism; osmoprotection and redox balance; and vitamin and cofactor biosynthesis. Selection was independent of pathway completeness or presence/absence patterns across the analyzed genomes. The predicted pathway completeness and presence/absence profiles were visualized as a heatmap using R version 3.2.0 with the *ComplexHeatmap*, *circlize*, and *grid* packages.

### 2.9. Statistical Analysis

Data are presented as the mean ± standard error (SE). The normality of model residuals was assessed using the Shapiro–Wilk test, and homogeneity of variance was evaluated using Levene’s test. No significant violations of these assumptions were detected. Differences among treatments were evaluated using one-way analysis of variance (ANOVA), followed by Fisher’s least significant difference (LSD) test. Differences were considered statistically significant at *p* < 0.05.

## 3. Results

### 3.1. Effects of C. amphilecti on Soybean Seed Germination and Root Development

Treatment with the cell-free culture supernatant increased soybean germination by only approximately 2% relative to the control, whereas viable *C. amphilecti* cells produced a significant increase of approximately 11% (Figure 1A). The treatments had more pronounced effects on early soybean root development. Root length reached approximately 1.6 and more than 2.3 times the control value following treatment with the supernatant and viable cells, respectively (Figure 1B). Treatment with the cell-free culture supernatant did not significantly affect root fresh weight, whereas viable *C. amphilecti* cells increased it approximately 1.8-fold relative to the control (Figure 1C).

Representative images were consistent with the quantitative measurements (Figure 1D). Supernatant-treated seedlings displayed visibly elongated but relatively sparse root systems. Seedlings treated with viable bacterial cells developed both longer and more extensive root systems than control seedlings.

### 3.2. Effect of C. amphilecti on the Productivity and Physio-Biochemical Responses of G. max

To determine whether KMM 296 persisted following the single seed treatment, culturable KMM 296 cells were recovered from soybean roots at both 20 and 80 days after sowing. Their abundance decreased from 2.6 × 10^4^ CFU g^−1^ FW at 20 days to 7.3 × 10^2^ CFU g^−1^ FW at 80 days, indicating that a culturable root-associated population was still detectable at the end of the experiment (Figure S2B). No KMM 296 colonies were recovered from plants treated with the medium control or cell-free culture supernatant.

Representative images of seeds harvested from plants in the different treatment groups are shown in Figure 2A. Inoculation with viable *C. amphilecti* cells significantly enhanced phytohormone accumulation in *G. max* tissues (Figure 2B). Root indole-3-acetic acid (IAA) concentration was approximately twice that in the control and supernatant-treated plants, while a similar but less pronounced upward trend was observed in the shoots. Gibberellin (GA) concentrations in the roots and shoots were approximately 2.5-fold and 1.3-fold higher than those of the control, respectively. For both hormones, no significant differences were observed between the control and supernatant treatments.

Treatment with viable *C. amphilecti* cells significantly increased plant height relative to the control, whereas the cell-free culture supernatant produced an intermediate response that did not differ significantly from either the control or viable-cell treatment (Figure 2B). Viable-cell treatment also increased the number of pods per plant by approximately 50%, while the supernatant did not significantly affect this parameter. The strongest responses were observed for seed production and total yield. The supernatant increased seed number and yield per plant by approximately 20% and 30%, respectively, whereas viable cells increased these parameters by approximately 96% and 136% relative to the control. For both seed number and yield, all three treatment groups differed significantly from one another (*p* < 0.05).

Inoculation with *C. amphilecti* cells also significantly enhanced photosynthetic pigment accumulation (Figure 2B). Chlorophyll a (Chla) and chlorophyll b (Chlb) contents in the bacterial treatment group were 64 and 44% higher than those in the control, and 22% and 20% higher than the values observed following the supernatant treatment. Furthermore, carotenoid content in plants treated with viable cells was more than 2.2-fold higher than in the control and approximately 1.4-fold higher than in the supernatant-treated group. In contrast, total phenolic content (TPC) did not differ significantly among the treatments.

### 3.3. Effect of C. amphilecti on Growth-Related Gene Expression in G. max

To assess the molecular responses associated with the growth-promoting effect of *C. amphilecti*, we analyzed the expression of six genes involved in auxin biosynthesis and signaling, gibberellin metabolism, cell wall modification, and root development. Their expression levels were comparable between control and supernatant-treated plants, whereas treatment with viable *C. amphilecti* cells was associated with changes in their expression (Figure 3).

The strongest response was observed for *GmYUC2a*, which encodes an enzyme involved in auxin biosynthesis. Its transcript level increased 4.97-fold relative to the control. Expression of the auxin-responsive transcription factor gene *GmARF8a* increased 1.75-fold, suggesting that bacterial treatment influenced genes associated with both auxin biosynthesis and signaling. The expression levels of the gibberellin metabolism-related genes *GmGA20ox1a* and *GmGA3ox1a* increased 1.58- and 2.71-fold, respectively. The transcript level of *GmEXPB2*, associated with cell wall modification and root elongation, also increased 1.81-fold. In contrast, the transcript level of *GmNIN1a*, which encodes a transcription factor involved in rhizobial infection and root nodule formation, was downregulated 4.41-fold relative to the control. The coordinated induction of auxin- and gibberellin-related genes together with *GmEXPB2* is consistent with the enhanced root development observed in bacteria-treated soybean plants.

### 3.4. Comparative Analysis of Predicted Metabolic Pathway Completeness in Cobetia genomes

Comparative reconstruction of 23 selected metabolic pathways across 34 *Cobetia* genomes showed broadly conserved predicted pathway profiles potentially relevant to plant–microbe interactions (Figure 4). The pathways were grouped into five functional categories: phytohormone and volatile-compound metabolism; phosphorus, nitrogen, and iron metabolism; polyamine metabolism; osmoprotection and redox balance; and vitamin and cofactor biosynthesis. Most strains showed high pathway completeness for indole-3-acetate biosynthesis V, L-tryptophan biosynthesis, acetoin and *meso*-butanediol formation, phosphate acquisition and transport, polyphosphate metabolism, putrescine biosynthesis, ectoine and glycine betaine production, and several cofactor-related pathways.

The metabolic profile of *C. amphilecti* KMM 296 was broadly consistent with this conserved, genus-wide pattern. Gapseq predicted complete indole-3-acetate biosynthesis V and L-tryptophan biosynthesis pathways in KMM 296, indicating potential genetic capacity for auxin-related metabolism rather than demonstrated auxin production. The pathways for pyruvate fermentation to (R)-acetoin I and meso-butanediol biosynthesis II were also predicted as complete. These predictions suggest potential for volatile-compound biosynthesis, but production of these metabolites and their contribution to the soybean responses were not experimentally assessed.

Several nutrient-acquisition pathways were predicted in strain KMM 296, including phosphate acquisition, phosphate ABC transporter, polyphosphate metabolism, and 2-aminoethylphosphonate degradation I. The first three were complete and highly conserved across the analyzed strains, whereas the 2-aminoethylphosphonate degradation pathway varied more among strains and was only partially complete in KMM 296. Ammonia assimilation cycle II was also predicted, while assimilatory nitrate reduction V and aerobactin biosynthesis were incomplete. No complete nitrogen fixation pathway was identified among the selected functions.

Gapseq also predicted pathways for putrescine and spermidine biosynthesis in KMM 296, suggesting potential genetic capacity for polyamine production. Complete pathways for ectoine, glycine betaine, trehalose, and glutathione biosynthesis further indicated an extensive capacity for adaptation to osmotic and oxidative stress. Most of these functions were conserved across the genus, although several strains lacked the selected trehalose biosynthesis pathways, indicating strain-dependent differences in carbohydrate-based osmoprotection.

Among the vitamin and cofactor pathways, tetrahydrofolate, pyridoxal 5′-phosphate, and biotin biosynthesis were predicted for KMM 296. In contrast, thiamine diphosphate biosynthesis I was incomplete and was therefore classified as absent under the applied *gapseq* criteria. The incomplete status of this and several other reconstructed pathways may reflect genuine differences in gene content, incomplete genome assemblies or annotations, or limitations of homology-based pathway reconstruction [27]. The latter possibility is particularly relevant to marine bacterial genomes, in which some functional genes may be annotated as hypothetical proteins because of limited similarity to characterized reference sequences and stringent alignment thresholds [28,29]. Consequently, annotation limitations may contribute to underestimated completeness of some non-conserved pathways, although this possibility cannot be distinguished from true gene absence without further validation.

Overall, the reconstructed metabolic profile suggests that KMM 296 possesses conserved metabolic potential related to nutrient acquisition, stress adaptation, and auxin-, volatile-compound-, and polyamine-associated metabolism. These in silico predictions are consistent with the observed stimulation of soybean root architecture and productivity and provide a genomic framework for further investigating the growth-promoting potential of KMM 296 as a euryhaline bioinoculant. However, the reconstructed pathways represent predicted metabolic capacities rather than direct evidence of transcription, enzyme activity, metabolite production, or the mechanisms underlying the observed plant responses.

## 4. Discussion

The present study demonstrates that the euryhaline marine bacterium *C. amphilecti* KMM 296 can substantially promote soybean development and productivity, with the magnitude of the response depending strongly on the form of bacterial treatment. The supernatant marginally affected germination, increased root length but not root fresh weight, and moderately enhanced seed number and yield, whereas viable cells significantly improved germination, root elongation, biomass accumulation, and reproductive performance (Figure 1 and Figure 2). Culturable KMM 296 cells were recovered from soybean roots, indicating prolonged persistence of a root-associated population after the single seed treatment. This finding supports the possibility that the stronger responses to the viable-cell treatment involved continued bacterial activity, including the production of growth-regulating compounds and metabolic responses to plant-derived substrates and signals [30,31]. However, the weaker effects of the cell-free supernatant do not by themselves confirm this interpretation, because bacterial growth alters both the nutrient composition and metabolite content of the culture medium. Accordingly, the comparison between the two preparations should not be interpreted as directly isolating the effects of cell viability from those of extracellular products. Nevertheless, the positive plant responses to the viable-cell preparation relative to the matched uninoculated-medium control support the growth-promoting potential of KMM 296.

The observed differences in soybean phytohormone levels and gene expression are consistent with the possibility that viable KMM 296 cells influence endogenous plant regulatory pathways rather than acting solely through the delivery of bacterial metabolites. The induction of *GmYUC2a*, which encodes a YUCCA-family enzyme involved in auxin biosynthesis, was consistent with the increased indole-3-acetic acid content in bacteria-treated roots, whereas *GmARF8a* upregulation suggests altered auxin-responsive signaling. Both genes have previously been implicated in the regulation of soybean root development and nodulation [32,33]. The complete indole-3-acetate biosynthesis V pathway predicted by gapseq (Figure 4) further suggests bacterial potential for auxin-related metabolism, although its activity and contribution to the measured IAA content remain unresolved. It should be emphasized that the metabolic pathways identified through genome-based reconstruction represent predicted genetic potential rather than confirmed metabolite production or pathway activity in situ. Moreover, although all samples within each tissue type were processed and analyzed under identical conditions, recovery, matrix effects, and antibody specificity were not independently validated for soybean extracts. Accordingly, the ELISA measurements support comparisons among treatments, but the absolute hormone concentrations should be interpreted in the context of this analytical limitation.

In plants treated with viable KMM 296 cells, the higher root GA content coincided with the induction of *GmGA20ox1a* and *GmGA3ox1a*, which encode enzymes involved in bioactive gibberellin biosynthesis [34]. Together, these observations are consistent with changes in plant GA metabolism but do not establish the relative contributions of plant and bacterial processes. Upregulation of *GmEXPB2*, an expansin gene associated with cell-wall modification and root elongation, may also be related to the observed root phenotype [35]. Notably, treatment with the supernatant increased root length without producing comparable changes in the analyzed genes. This difference may indicate that preformed extracellular metabolites transiently promoted elongation, whereas viable cells elicited a broader and more sustained physiological response. This observation does not exclude transcriptional responses involving other genes or occurring at other time points.

Unlike the other analyzed genes, *GmNIN1a* was significantly downregulated following treatment with viable *C. amphilecti* cells. NIN transcription factors are central regulators of rhizobial infection and nodule organogenesis in legumes [36]. Therefore, the observed expression pattern differs from the canonical response to compatible nitrogen-fixing symbionts and suggests that the growth-promoting effect of KMM 296 does not involve activation of the typical nodulation program. Consistent with this interpretation, no complete nitrogen-fixation pathway was identified in the analyzed *Cobetia* genomes (Figure 4), indicating that the observed effects are unlikely to result from bacterial nitrogen fixation. Given the substantial carbon and energy requirements of nodulation [37], one hypothesis is that reduced *GmNIN1a* expression reflects a regulatory balance favoring vegetative root development over the activation of symbiotic signaling pathways. However, nodulation and other components of symbiotic signaling were not assessed, and this hypothesis remains to be tested.

The enhanced root system was accompanied by increased plant height and improved yield components, including pod number, seed number, and seed yield per plant. Improved resource acquisition by a larger root system may support shoot growth and reproduction, while the increased photosynthetic pigment levels may reflect improved physiological status. This pattern agrees with previous soybean studies in which selected bacterial inoculants increased pod or grain production, although their effects on chlorophyll content and photosynthetic traits varied among bacterial strains and experimental conditions [1,2,3]. Total phenolic content remained unchanged, indicating that bacterial treatment did not increase the overall accumulation of phenolic compounds under the experimental conditions.

Genome-based reconstruction predicted high completeness of the indole-3-acetate and L-tryptophan biosynthesis pathways, suggesting potential genetic capacity for auxin-related metabolism in KMM 296, as L-tryptophan is a precursor of bacterial IAA [38]. Predicted pathways for acetoin and meso-2,3-butanediol biosynthesis are also of interest. Bacterial volatile compounds can modify root system architecture, and acetoin and 2,3-butanediol have been directly implicated in plant growth promotion [39,40]. Their continued production could partly explain the stronger effects of viable cells, although their production by KMM 296 remains to be confirmed.

Predicted pathways related to phosphate acquisition and metabolism may also be relevant. KMM 296 has previously been reported to produce an active extracellular alkaline phosphatase [41], supporting the potential importance of phosphorus turnover in the physiology of this strain. Extracellular phosphatase activity could release inorganic phosphate from organic phosphorus compounds, whereas the predicted pathways for high-affinity phosphate transport and polyphosphate metabolism are more likely to support bacterial phosphate acquisition, storage, and homeostasis under phosphorus-limited conditions [42,43]. Although phosphate-solubilizing rhizobacteria can alter rhizosphere phosphorus availability and root traits [44], the contribution of the predicted KMM 296 functions to the observed soybean response remains untested. Incomplete nitrate-reduction and aerobactin-biosynthesis pathways, together with the absence of a complete nitrogen-fixation pathway, provide no genomic support for these mechanisms.

Predicted putrescine and spermidine biosynthesis pathways suggest a potential for polyamine production, which may contribute to growth regulation and stress responses during plant–bacterium interactions [45]. However, some polyamine routes were supported by a limited number of key reactions and therefore require biochemical validation. Complete pathways for ectoine, glycine betaine, and trehalose biosynthesis are more likely to support bacterial osmotic adaptation [46], whereas glutathione biosynthesis may contribute to redox homeostasis [47], potentially facilitating bacterial survival during seed treatment and in the plant-associated environment. Vitamin and cofactor biosynthesis likewise reflects bacterial metabolic competence rather than evidence of direct vitamin transfer to the plant. Notably, most predicted functions were broadly conserved across the analyzed *Cobetia* genomes, suggesting a shared functional repertoire related to auxin metabolism, phosphate acquisition, polyamine biosynthesis, and osmotic adaptation. Conversely, variation in the completeness of 2-aminoethylphosphonate degradation I and trehalose biosynthesis indicates potential strain-level functional differentiation.

Taken together, the phenotypic, physiological, transcriptional, and comparative genomic data suggest that KMM 296-induced growth promotion may involve several complementary bacterial and plant-mediated mechanisms. However, the genomic predictions describe potential metabolic capacities rather than demonstrated pathway activity and should not be considered evidence of the mechanisms underlying the soybean responses. The relative contributions of bacterial and plant-mediated processes remain to be experimentally resolved.

## 5. Conclusions

This study demonstrates the plant growth-promoting potential of the euryhaline marine bacterium *Cobetia amphilecti* KMM 296 in soybean. Treatment with viable bacterial cells enhanced seed germination, root development, plant growth, photosynthetic pigment levels, seed production, and yield. The cell-free culture supernatant produced weaker or intermediate responses and did not reproduce the full range or magnitude of effects observed with the viable-cell preparation under the tested conditions.

Enhanced root growth was accompanied by increased auxin and gibberellic acid levels and altered expression of genes involved in hormone biosynthesis and signaling, cell-wall modification, and root development. Comparative genomic analysis predicted metabolic potential related to auxin and volatile-metabolite production, phosphate acquisition, polyamine biosynthesis, and adaptation to osmotic and oxidative stress. However, these genomic predictions require biochemical and functional validation.

KMM 296 therefore represents a promising marine-derived candidate for soybean bioinoculant development. Further studies should examine root colonization, metabolite production, interactions with rhizobia, and inoculant performance under diverse soil, field, and abiotic stress conditions.

## Figures and Tables

**Figure 1:**
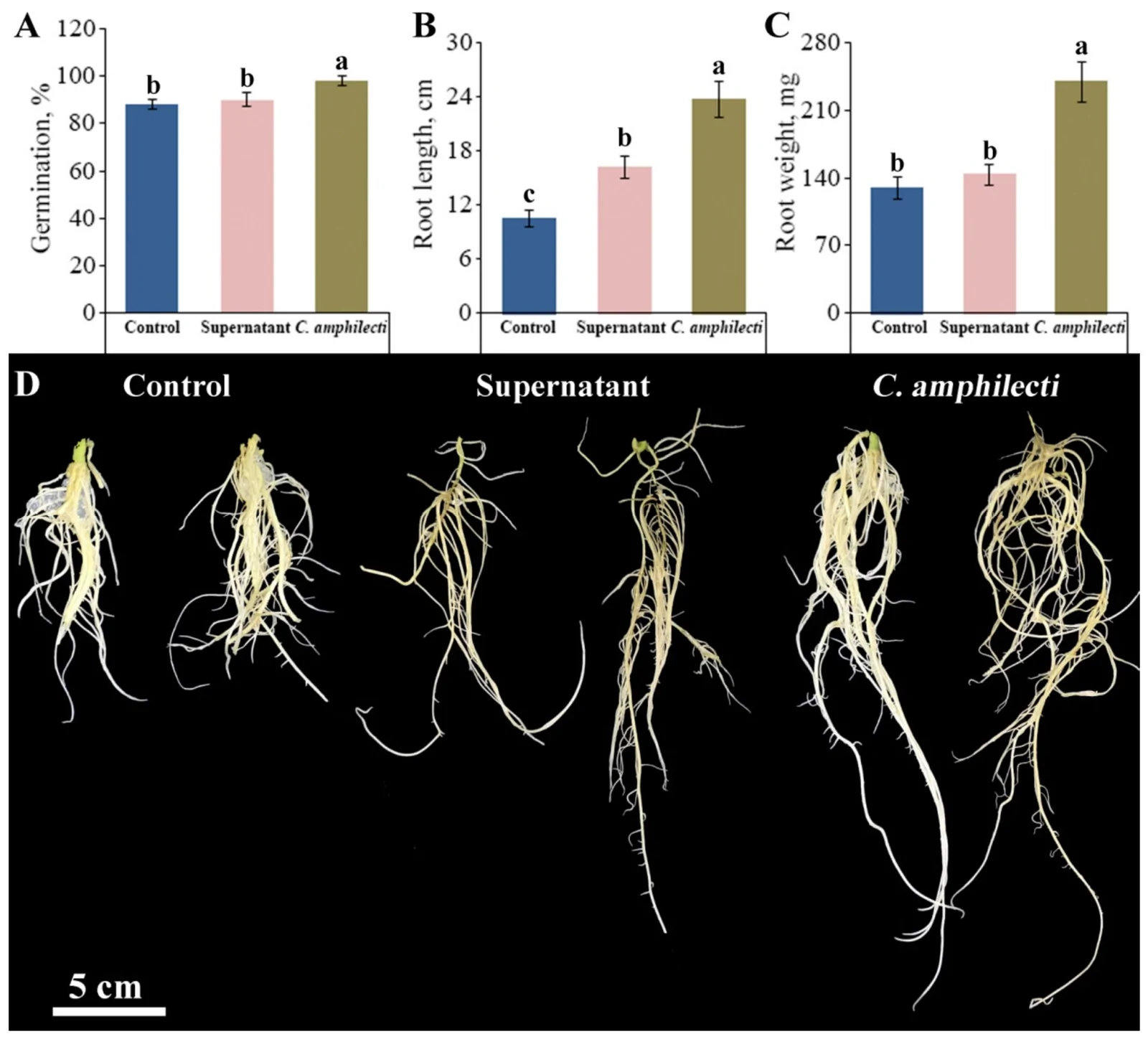
Effects of cell-free culture supernatant and *C. amphilecti* cells on soybean seed germination and root development. (**A**) Germination percentage. (**B**) Root length. (**C**) Root fresh weight. (**D**) Representative root systems of seedlings. Data are presented as mean ± standard error. Different letters above the bars indicate statistically significant differences among treatments (*p* < 0.05, ANOVA followed by Fisher’s LSD test).

**Figure 2:**
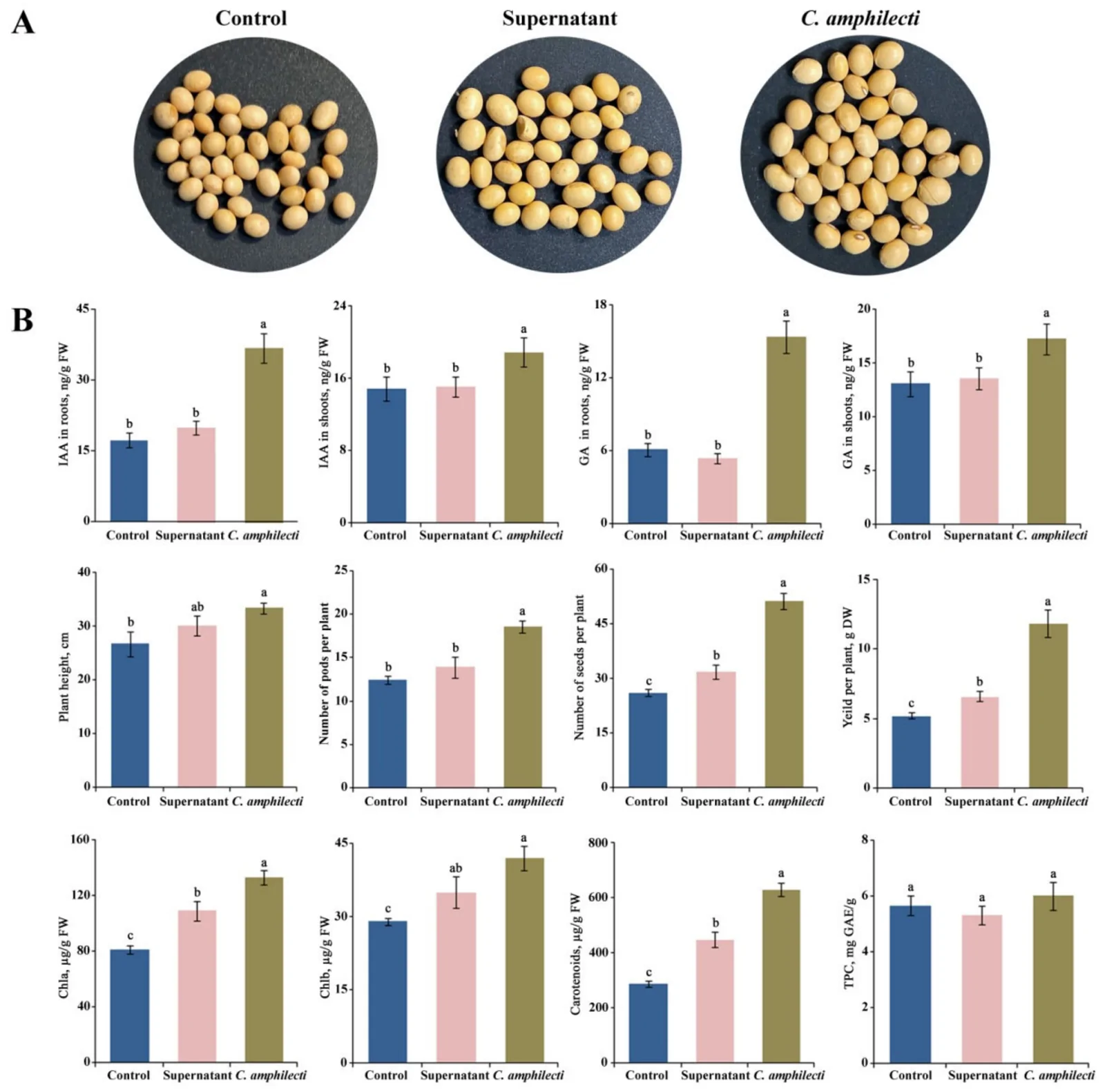
Effect of *C. amphilecti* treatment on the productivity, phytohormone content, and physiological and biochemical parameters of *G. max*. (**A**) Seed appearance. (**B**) Indole-3-acetic acid (IAA) and gibberellin (GA) content in roots and shoots, plant height, number of pods and seeds per plant, yield per plant, Chla, Chlb, carotenoids, and total phenolic compounds (TPC) content. Data are presented as mean ± standard error. Different letters above the bars indicate statistically significant differences between treatments (*p* < 0.05; one-way ANOVA followed by Fisher’s LSD test).

**Figure 3:**
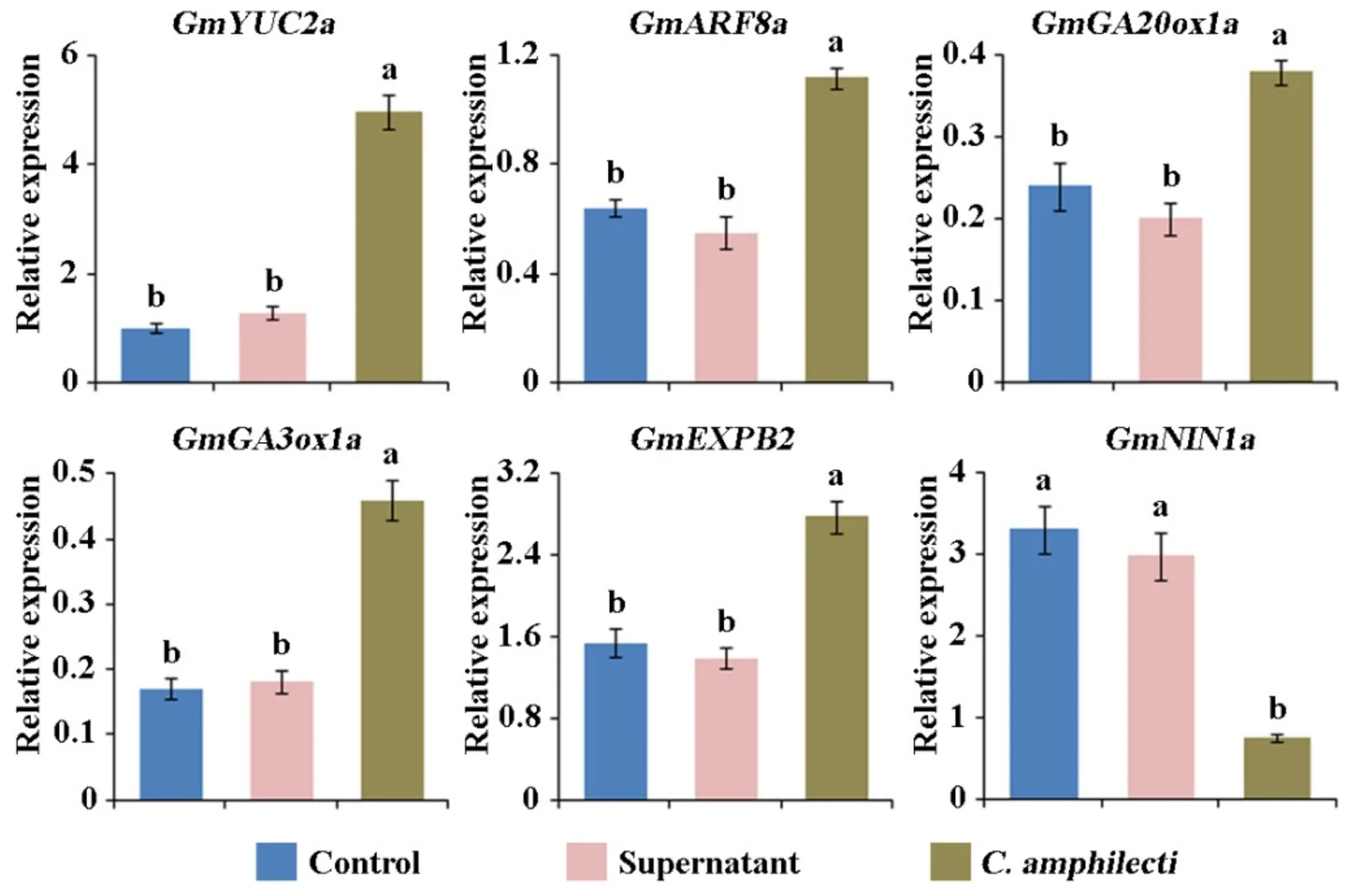
Expression of selected genes in soybean roots under control, cell-free supernatant, and viable *C. amphilecti* KMM 296 treatments. Data are presented as mean ± standard error. Different letters above the bars indicate statistically significant differences between treatments (*p* < 0.05; one-way ANOVA followed by Fisher’s LSD test).

**Figure 4:**
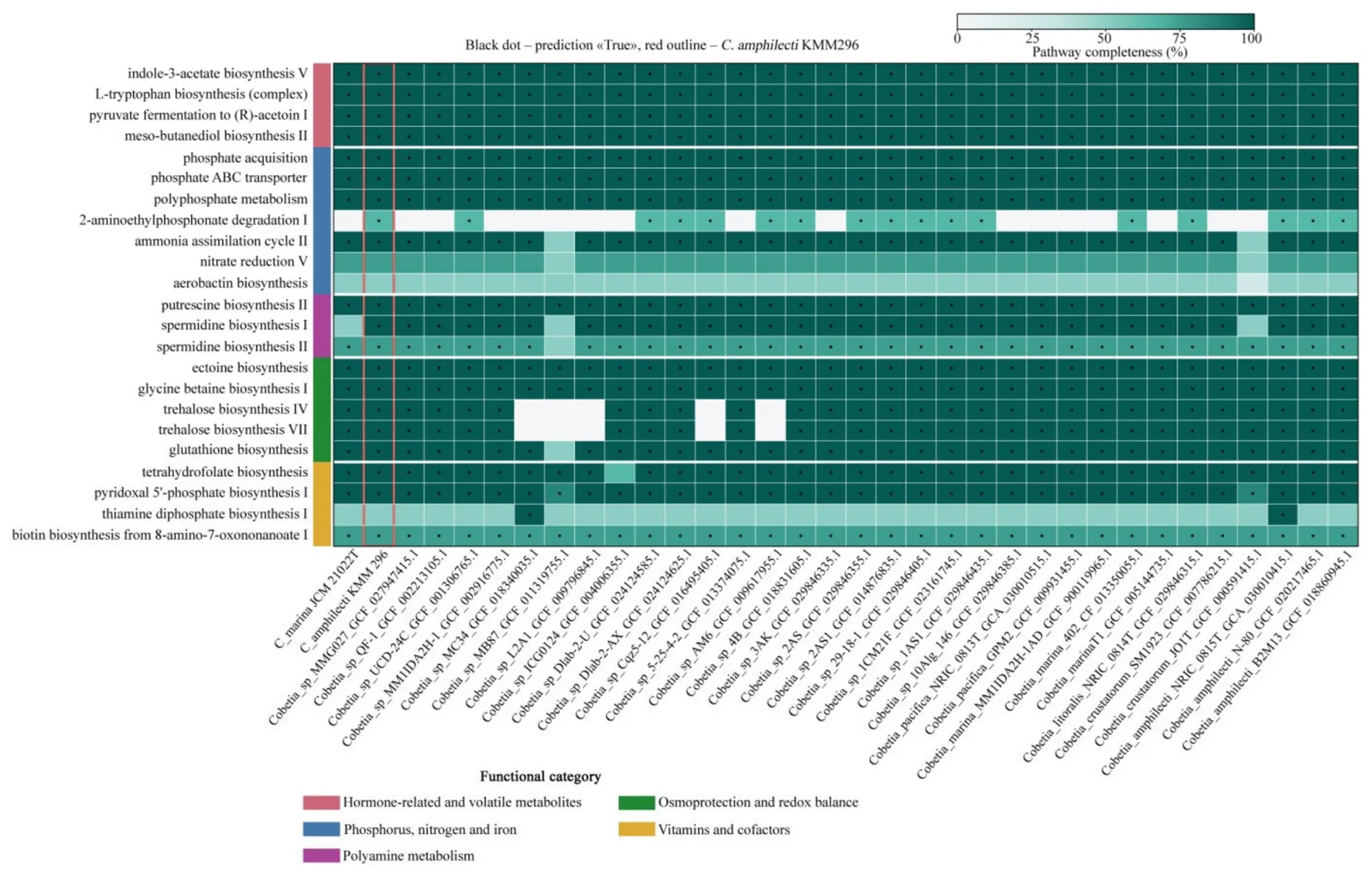
Predicted completeness and functional classification of 23 selected metabolic pathways across 34 *Cobetia* genomes. RefSeq accession numbers for all analyzed genomes are provided as column labels. Rows represent metabolic pathways, and columns represent individual genomes. Cell color indicates predicted pathway completeness (0–100%). Black dots denote pathways classified as present (“True”) by gapseq, and colored bars on the left indicate functional categories. The *C. amphilecti* KMM 296 genome is outlined in red.

## Data Availability

The original contributions presented in this study are included in the article/Supplementary Material. Further inquiries can be directed to the corresponding authors.

## Supplementary Materials

The following supporting information can be downloaded at: https://www.mdpi.com/article/10.3390/plants15203221/s1, Table S1: List of primer sequences used in this study. Figure S1. Expression stability analysis of candidate reference genes in *G. max*, as evaluated using RefFinder. Figure S2. Persistence and molecular confirmation of culturable *C. amphilecti* KMM 296 associated with soybean roots.
