# Supplementary material for: Soybean Growth Promotion by the Euryhaline Marine Bacterium Cobetia amphilecti KMM 296 Is Associated with Hormonal and Transcriptional Responses
Source: Plants (Basel). 2026 Oct 20;15(20):3221. doi: 10.3390/plants15203221 (PMC13708865; doi:10.3390/plants15203221)
Supplement: Supplementary file 1 [file plants-15-03221-s001.zip › plants-4546247-supplementary.pdf]

Supplementary Materials

Soybean growth promotion by the euryhaline marine bacterium *Cobetia amphilecti* KMM 296 is associated with hormonal and transcriptional responses

Yulia A. Yugay [1]*, Andrey A. Sadykov [1], Olesya D. Kudinova [1], Yulia L. Yaroshenko [1], Alexandra V. Fialko [1], Maria R. Sorokina [1], Egor S. Alaverdov [1], Elena A. Vasyutkina [1], Tatiana V. Rusapetova [1], Olga I. Nedashkovskaya [2], Victor P. Bulgakov [1], Larissa A. Balabanova [2]* and Yury N. Shkryl [1]*

[1] Federal Scientific Center of the East Asia Terrestrial Biodiversity, Far East Branch of Russian Academy of Sciences, Vladivostok, 690022, Russia
[2] G.B. Elyakov Pacific Institute of Bioorganic Chemistry, Far Eastern Branch, Russian Academy of Sciences, Vladivostok, 690022, Russia

* Correspondence: yuya1992@mail.ru; lbalabanova1@gmail.com; yn80@mail.ru

Table S1. Primer sequences and qPCR amplification parameters used in this study.

Gene (GenBank Acc. No.)	Forward primer	Reverse primer	Efficiency (%)	R^2
		Glycine max		
GmELF1B (NM_001249608)	AAGTGTATGCAGCTGTTGTTG	TCTTACCCCTTGAGCGTGG	96.91	0.981
GmCons6 (NM_001255441)	GTATCCACTTTTGCGTCCAG	CACTGTAGCTAGAAAAGGATC	98.14	0.988
GmCYP2 (NM_001357079)	CCCGAACTTCATGTGCCAG	GGGACCAGTGTGCTTCTTC	101.36	0.990
GmACT (NM_001289231)	GCACCCAGCAGCATGAAGA	GTCCATCAATTGTGCACAGAA	95.10	0.989
GmTUB (NM_001250372)	GACCACAAGTTTGATCTGATG	TCCTCTCCTTCCTCATCATC	95.94	0.991
GmYUC2a (XM_003524344)	TGTGATCATTCTAGCAACTGG	GATGCACCAAGAAGGCCAC	100.52	0.984
GmARF8a (XM_041008615)	AGCCACAAGCCCAAGTAATG	GAAGGTAGACCAGAGTGTTG	96.19	0.992
GmEXPB2 (NM_001255271)	TTGTGGTTATGGAAGTGCTGT	CACACCCAGGACAGCTATC	97.48	0.997
GmGA20ox1a (XM_003522517)	CCATGACAACACCTTAGAGC	CCACGCCTAAGCTCATTGC	97.41	0.991
GmGA3ox1a (XM_003546191)	TGTTGCTCATGTTGGATTCTC	TGGTAAAGGATTGTGAGAAGG	100.47	0.997
GmNIN1a (XM_041014704)	ACGAGATTAGCAACATTCAGC	AGAGGCGAAGCTTGAAAGAG	98.66	0.983
		Cobetia amphilecti		
CaAP (DQ435608)	CAGAGATCAAGAATGTCATTC	CTTCGCTACCACTGTCTTCAG	n.a.	n.a.

n.a., not applicable; the *CaAP* primer pair was used for endpoint colony PCR.

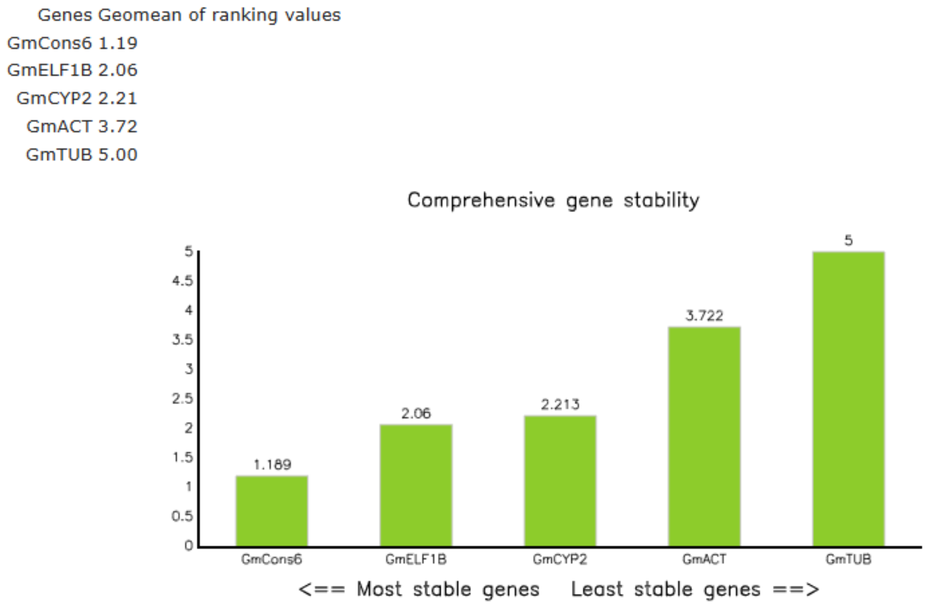

Figure S1. Expression stability analysis of candidate reference genes in *G. max*, as evaluated using RefFinder. The bar graph presents the comprehensive ranking calculated as the geometric mean of the rankings obtained with four algorithms: geNorm, NormFinder, BestKeeper, and the comparative ΔCt method. Lower values indicate greater expression stability. Gene abbreviations: *GmACT*, actin; *GmTUB*, β-tubulin; *GmCons6*, F-box protein family member; *GmELF1B*, translation elongation factor 1-beta; *GmCYP2*, cyclophilin 2.

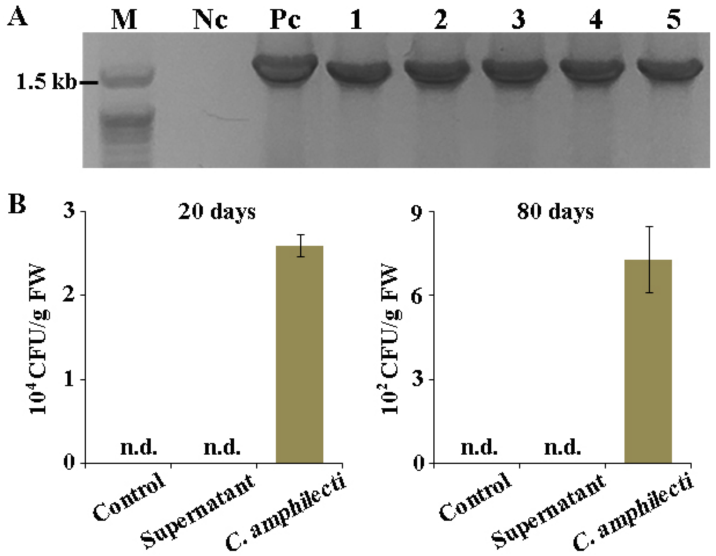

Figure S2. Persistence and molecular confirmation of culturable *C. amphilecti* KMM 296 associated with soybean roots. (A) Colony PCR verification of presumptive KMM 296 colonies recovered from soybean roots. Amplification of the *CaAP* gene marker supported their identification as KMM 296. Lanes 1–5, colonies randomly selected from independent plates; Pc, positive control containing genomic DNA from a pure KMM 296 culture; Nc, no-template negative control; M, DNA size marker. (B) Abundance of culturable root-associated KMM 296 cells at 20 and 80 days after sowing, expressed as CFU per gram of fresh root weight. Data are presented as mean ± standard error. n.d., not detected.
